# Embolisation of an aneurysmal high-flow renal arteriovenous fistula in a paediatric patient: simultaneous arterial and venous approach

**DOI:** 10.1186/s42155-022-00303-4

**Published:** 2022-05-27

**Authors:** Kin Fen Kevin Fung, Sze Wah Wong, Eugene Yu-hin Chan, Ka-king Cheng, Hing-Yan Cho, Elaine Yee-Ling Kan, Alison Lap Tak Ma

**Affiliations:** 1 Department of Radiology, Hong Kong Children’s Hospital, Hong Kong, Hong Kong SAR; 2 Paediatric Nephrology Centre, Hong Kong Children’s Hospital, Hong Kong, Hong Kong SAR; 3 Department of Diagnostic and Interventional Radiology, Kwong Wah Hospital, Hong Kong, Hong Kong SAR

**Keywords:** Embolisation, Renal arteriovenous fistula, Embolic migration, Coil, N-butyl cyanoacrylate

## Abstract

**Background:**

A large aneurysmal renal arteriovenous fistula (AVF) can cause hypokalaemic hypertension due to activation of renin-aldosterone system due to steal effect from renal parenchyma. In comparison to nephrectomy, endovascular embolisation of renal AVF is minimally invasive and can be nephron sparing, thus preserving renal function. However, such embolisation is technically challenging and can be associated with high risk of embolic migration.

**Case presentation:**

We present a case of successful embolisation of a large aneurysmal renal AVF in a 11-year-old girl. The AVF was initially treated with coil embolization via transarterial route, resulting in partial migration of coil into inferior vena cava. After removal of the migrated coil via transvenous snaring, coils were deployed simultaneously via transarterial and transvenous routes to prevent migration. AVF flow dampened but residual flow persisted at 1 month follow up. A second embolization session with additional coil deployment and N-butyl cyanoacrylate (NBCA) injection resulted in successful occlusion of the AVF. At 3 months follow up, the girl’s blood pressure and serum potassium level have normalized without need of antihypertensive agent or potassium supplements.

**Conclusion:**

Endovascular embolisation can be an effective nephron sparing treatment for large aneurysmal renal AVF. This is particularly important in paediatric patients as most renal function can be preserved with their expected longer life span. Risk of coil migration can be controlled by simultaneous transarterial and transvenous deployment. Complete occlusion of AVF can be aided by additional use of NBCA.

## Background

Large high flow renal arteriovenous fistula (AVF) can present with hypertension due to steal effect from renal parenchyma and subsequent activation of renin-aldosterone system (An et al. 2009). Endovascular embolization of renal AVF is a minimally invasive and organ preserving treatment when compared to nephrectomy. However, such embolisation is technically challenging and can be associated with high risk of embolic migration. In this report, we share the embolization strategies of a high flow aneurysmal renal AVF in a 11-year-old girl.

## Case presentation

**Fig. 1:**
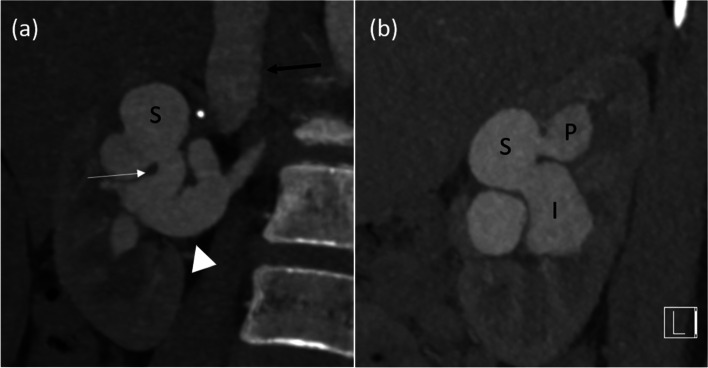
Selected maximum intensity projection arterial phase contrast enhanced CT images performed at diagnosis. **a** Coronal reformatted image showed an aneurysmal AVF at right renal hilum. The fistula (white arrow) connects an aneurysmally dilated anterior division of right renal artery (white arrowhead) with the superior venous varix (marked with “S”). Early opacification of IVC detected on arterial phase, indicating high flow shunting. **b** Sagittal reformatted image demonstrated the three interconnecting venous varices – superior (marked as “S”), inferior (marked as “I”) and posterior (marked as “P”)

**Fig. 2:**
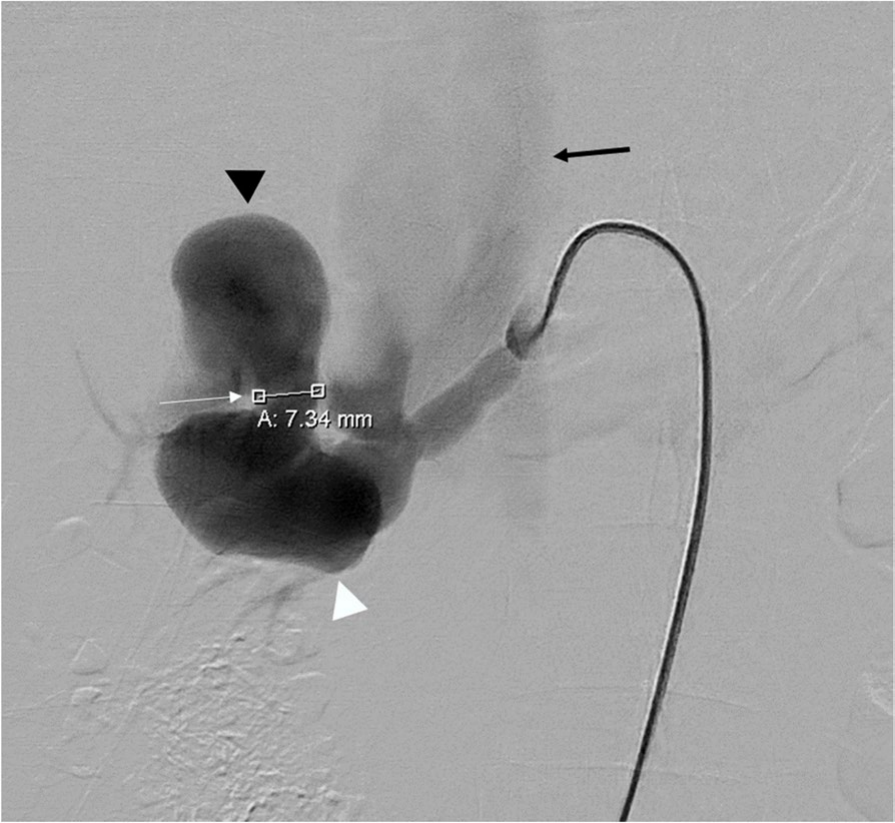
Selected image from DSA demonstrated a high flow aneurysmal AVF at right renal hilum. The fistula (white arrow) measures 7.34 mm and connects an aneurysmally dilated anterior division of right renal artery (white arrowhead) with the superior venous varix (black arrowhead). The IVC (black arrow) was dilated and opacified early, with impaired renal parenchymal staining, indicating rapid high flow arteriovenous shunting

An 11-year-old girl with good past health was referred to our centre because of hypokalaemic hypertension, with blood pressure up to 160/90mmHg. She did not have family history of reno-vascular disease nor history of previous renal biopsy or trauma. Physical examination revealed a loud para-umbilical bruit. Echocardiography showed normal ejection fraction with no evidence of left ventricular hypertrophy. Biochemical blood tests showed hypokalemia (3.1 mmol/L; laboratory reference range: 3.3–4.6 mmol/L) and secondary hyperaldosteronism with markedly elevated renin (11.96 ng/ml/hour; laboratory reference0 range 0.50–3.30 ng/ml/hour) & aldosterone (1208 pmol/L; laboratory reference range: = < 583 pmol/L) levels. Contrast enhanced computed tomography (CT) of abdomen and pelvis, and subsequently, digital subtraction angiography (DSA) were performed, showing a large high flow AVF involving the hilum of right kidney, with aneurysmal dilatation of main renal artery and several venous varices at right upper renal pole. The fistulous segment was very short, connecting an aneurysmally dilated anterior division of right renal artery and three interconnecting venous varices (superior, posterior and inferior). The superior venous varix then drained into a dilated inferior vena cava (IVC) (Figs. 1 and 2).

**Fig. 3:**
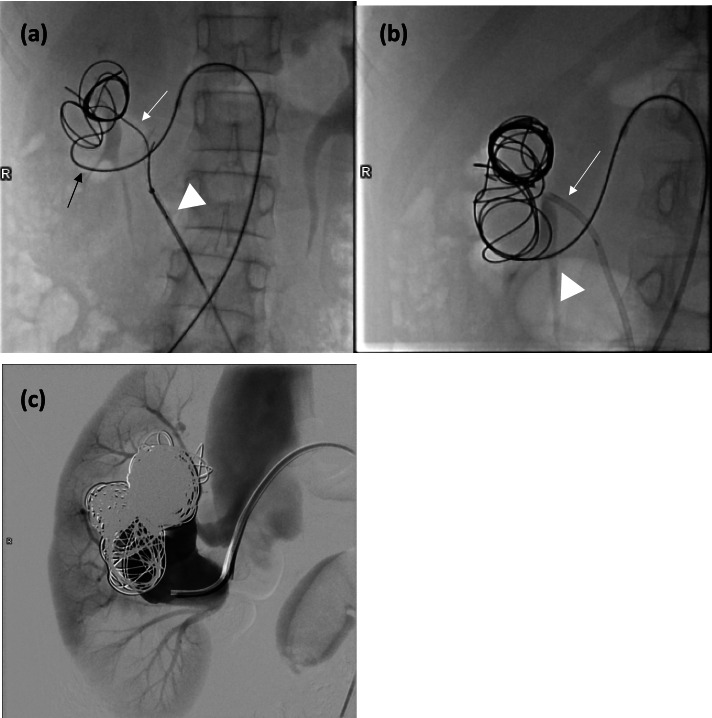
**a** During transarterial deployment of second 40 mm × 60 cm Ruby framing coil (black arrow), the first 40 mm × 60 cm Ruby framing coil (white arrow) unraveled and prolapsed into the IVC. It was then removed by a snaring catheter (white arrowhead) via transvenous route. **b** Two 40 mm × 60 cm Ruby framing coils were simultaneously deployed via transarterial and transvenous microcatheters to prevent coil migration (transarterial coil – white arrow; transvenus coil – white arrowhead). **c** Post-coiling angiogram showed improved parenchymal enhancement of right kidney but residual shunting into IVC along the AVF

Through a 6Fr vascular sheath via a right common femoral artery access, the right renal artery was cannulated with a 6Fr Mach 1 LIMA guiding catheter (BostonScientific, Marlborough, MA, USA). The superior venous varix was cannulated with a 2.6Fr PXSlim microcatheter (Penumbra, Alameda, CA, USA). Due to the complex interconnecting venous variceal anatomy, although the superior venous varix only measured 2.1 cm × 1.8 cm, the largest available framing Ruby coil (Penumbra, Alameda, CA, USA), i.e. 40 mm × 60 cm, was chosen and deployed. However, upon deployment of the second 40 mm × 60 cm Ruby framing coil, the first framing coil unravelled with its distal portion protruding into infrarenal IVC (Fig. 3a). A 6Fr vascular sheath was immediately placed into left common femoral vein (CFV) and the first framing coil was snared and removed using One Snare catheter (Merit Medical, Salt Lake City, UT, USA). The venous side was then cannulated with a 6Fr Mach 1 RDC guiding catheter (BostonScientific, Marlborough, MA, USA) and Renegade Hi-flo microcatheter (BostonScientific, Marlborough, MA, USA). In view of high risk of coil migration due to high flow within the AVF, two 40 mm × 60 cm Ruby framing coils were deployed via the transarterial and transvenous microcatheters simultaneously to allow entanglement of the coils and better anchoring in the superior venous varix (Fig. 3b). After achieving a stable coil mass, multiple (up to nineteen) framing Ruby coils and POD packing coils (Penumbra, Alameda, CA, USA) were deployed via the transarterial microcatheter. Check angiogram showed dampening of flow into IVC and improved parenchymal enhancement (Fig. 3c). At that juncture, we decided to conclude the procedure and observe if the fistula would spontaneously obliterate due to reduced flow after coil embolization.

**Fig. 4:**
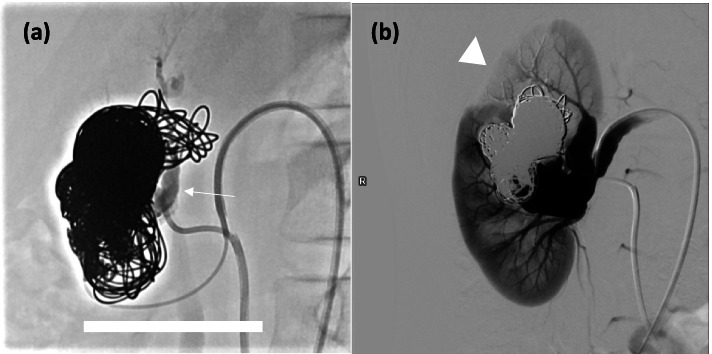
**a** The arterial supply of AVF was cannulated using a Swift-ninja mC and injection of 50% NBCA:lipiodol mixture was performed under fluoroscopic screening. The glue cast was indicated by white arrow. **b** Check angiogram showed complete occlusion of AVF with no residual shunting into IVC. Most of the renal parenchymal arterial branches were preserved. The anterosuperior segmental branch (arrowhead) was sacrificed as it shared a common origin with the arterial supply of AVF

One month after first embolization procedure, the girl still required oral potassium supplement and two antihypertensive medications. Reassessment angiogram showed persistent shunting along the right renal AVF. The superior venous varix was cannulated using PXslim microcatheter via 6Fr vascular sheath at left CFV. Further coil embolisation into the superior venous varix was performed with nine Ruby packing coils. However residual flow was still observed after achieving dense coil packing. Therefore, decision was made to proceed with NBCA embolization via transarterial route. The arterial supply of AVF, which shared a same origin as the anterosuperior segmental artery of right kidney, was cannulated using a Swift-ninja microcatheter (Merit Medical, Salt Lake City, UT, USA). 2 mL of 50% NBCA:lipiodol mixture was injected under fluoroscopic screening (Fig. 4a). Check angiogram showed complete obliteration of AVF with no residual shunting into IVC observed (Fig. 4b).

Follow up contrast enhanced CT performed 3 months post-embolisation showed persistent occlusion of the fistula. The girl’s blood pressure and serum potassium level have normalized. She no longer required anti-hypertensive medication or oral potassium supplements.

## Discussion

The aim of successful endovascular treatment of renal AVF involves complete and permanent occlusion of the fistula while preserving as much normal renal arterial branches as possible (Maruno et al. 2016). In a large aneurysmal high flow renal AVF such as our case, the technical challenges lie in several aspects.

Firstly, a short fistulous segment in our case necessitated precise deployment of coil to prevent inadvertent embolization of normal parenchymal arteries. We chose detachable coils to allow occlusion of shunt as closely as possible to the fistulous point. Vascular plug is a potential alternative (Campbell et al. 2009), however, the tortuous vascular anatomy in our case precluded the use of plug.

Secondly, the high flow within the fistula increases the risk of coil migration, as observed in our case where the first framing coil unravelled and prolapsed into IVC. To achieve a stable coil mass position, we deployed the coils simultaneously via transarterial and transvenous route to allow entanglement of coils and attainment of high coil volume within the fistula as quickly as possible. Other strategies to prevent coil migration include use of flow control balloon catheter and simultaneous transarterial coil deployment with dual microcatheters technique (Maruno et al. 2016; Greben et al. 2010). However, to adopt these techniques, a larger guiding catheter (up to 8Fr) and therefore femoral arterial sheath would be needed, which is not preferable in a paediatric patient. Alternatively access of contralateral common femoral artery might be needed to allow passage of an additional balloon catheter or guiding catheter. Balloon catheter may also be placed on venous side to prevent coil migration into IVC, however we did not adopt this technique with concern that balloon occlusion of venous outflow may increase pressure inside the AVF and potentially cause rupture (Maruno et al. 2016).

Thirdly, as illustrated in our case, the sole use of coil may not be sufficient to achieve total occlusion of the AVF. Additional use of high concentration NBCA was required to seal off the shunt. NBCA is a liquid adhesive that polymerises instantaneously upon contact with anions in blood. By mixing NBCA with lipiodol at a high concentration, this allows rapid polymerization of NBCA which can prevent migration (Takeuchi et al. 2014). The pre-existing densely packed coil mass also acts as a scaffold to prevent inadvertent NBCA migration without the need of additional flow arrest technique.

## Conclusion

Endovascular embolisation can be an effective nephron sparing treatment for large aneurysmal renal AVF. This is particularly important in paediatric patients as most renal function can be preserved with their expected longer life span. Risk of coil migration can be controlled by simultaneous transarterial and transvenous deployment. Complete occlusion of AVF can be aided by additional use of NBCA.

## Data Availability

Not applicable.

## Abbreviations

AVF: Arteriovenous fistula
CFV: Common femoral vein
CT: Computed tomography
IVC: Inferior vena cava
NBCA: N-butyl cyanoacrylate
LIMA: Left internal mammary
RDC: Renal double curve
